# P-1803. Why We Do What We Do: A Survey of ID Providers About Oral Antibiotics

**DOI:** 10.1093/ofid/ofae631.1966

**Published:** 2025-01-29

**Authors:** Lucy S Witt, Sujit Suchindran, Marisa Winkler

**Affiliations:** Emory University, Atlanta, Georgia; Emory University School of Medicine, Atlanta, GA; Element Materials Technology/Jones Microbiology Institute, North Liberty, Iowa

## Abstract

**Background:**

Multiple studies have shown the efficacy of oral antibiotics for serious infections. Despite this, many infectious disease (ID) providers continue to solely use intravenous (IV) antibiotics. To understand current practices among ID providers in our healthcare system, we conducted a survey to observe practice patterns, comfort level, and beliefs around oral antibiotics.

**Table 1.**
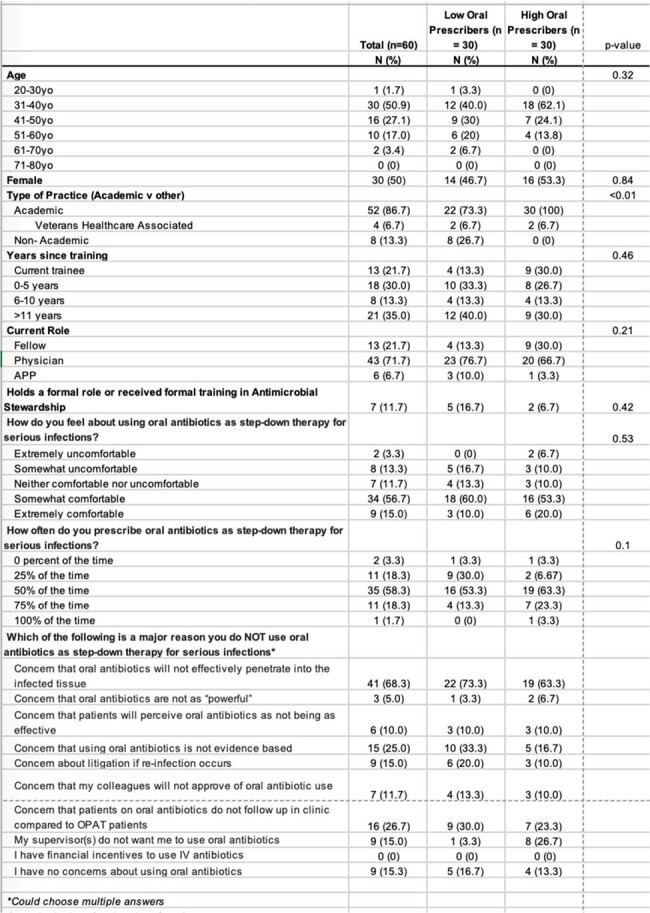
Respondent Demographics and Responses

**Methods:**

Our survey was distributed via email to 137 local ID providers at nine hospitals. The survey included questions about demographics, practice characteristics, and comfort with as well as barriers to prescribing oral antibiotics. Also included were five clinical cases for which respondents were asked to choose definitive therapy from a selection of IV and oral options. Based on responses, providers were classified as either high- or low-oral antibiotic prescribers (HOAP or LOAP). Categorical variables were analyzed using chi-square or Fischer’s exact test as appropriate.

**Table 2.**
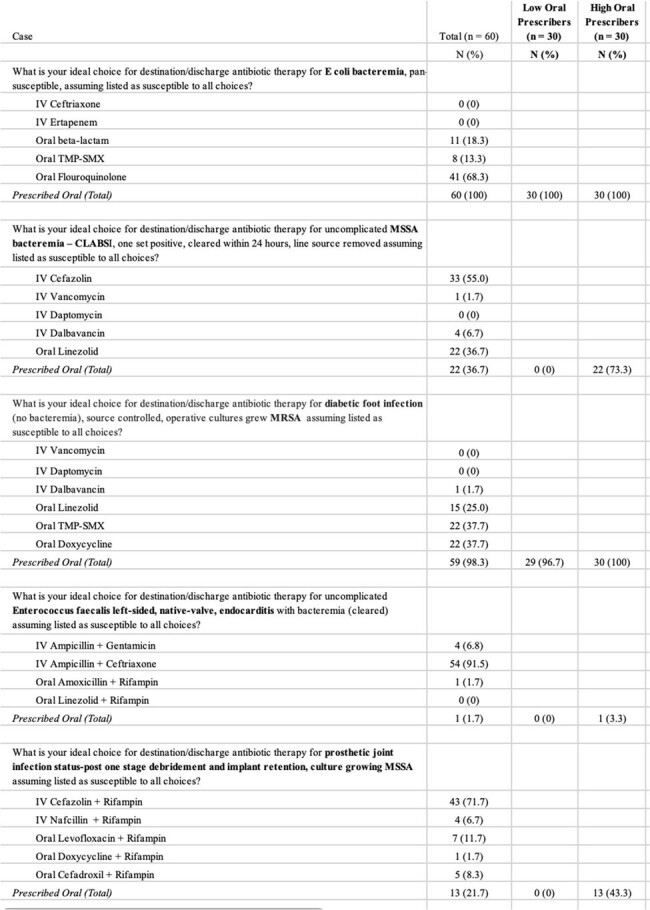
Respondent Answers to Clinical Cases

**Results:**

A total of 60 clinicians (44%) completed our survey. 87% were academic affiliated (52/60), while 13.3% (8/60) identified as private practice (Table 1). Half of respondents were categorized as LOAP. All respondents chose an oral step-down therapy for a case of *E. coli* bacteremia, while only one provider chose an oral option for step-down therapy for left-sided native valve endocarditis caused by *E. faecalis* (Table 2). 73.3% of HOAP chose oral antibiotics as definitive therapy for the case of an uncomplicated MSSA central line associated bloodstream infection compared to no LOAP (Table 2). The only statistically significant predictor of LOAP was type of practice; with all private practice providers classified as LOAP. HOAP were more likely to cite supervisor concerns with oral antibiotics as a barrier to prescribing oral antibiotics. Interestingly, similar numbers of HOAP and LOAP expressed they had no concerns about prescribing oral antibiotics.

**Conclusion:**

In this survey of ID providers regarding oral antibiotics, most demographics did not predict HOAP or LOAP. Certain clinical scenarios, such as Gram-negative bacteremia, led to oral antibiotic use in both LOAP and HOAP. This information can be used by stewardship programs to target interventions to encourage oral antibiotic step-down therapy in LOAP.

**Disclosures:**

**Marisa Winkler, MD, PhD**, Element Iowa City (JMI Laboratories) was contracted to perform services in 2023 for > 30 biotech and pharmaceutical companies: Grant/Research Support

